# Organic Cocrystals: Recent Advances and Perspectives for Electronic and Magnetic Applications

**DOI:** 10.3389/fchem.2021.764628

**Published:** 2021-12-09

**Authors:** Mengjia Jiang, Chun Zhen, Shuyu Li, Xiaotao Zhang, Wenping Hu

**Affiliations:** ^1^ Tianjin Key Laboratory of Molecular Optoelectronic Science, Department of Chemistry, School of Science, Tianjin University, Tianjin, China; ^2^ Institute of Molecular Aggregation Science, Tianjin University, Tianjin, China; ^3^ School of Chemistry and Chemical Engineering, Qinghai Minzu University, Qinghai, China; ^4^ Joint School of National University of Singapore and Tianjin University, International Campus of Tianjin University, Fuzhou, China

**Keywords:** cocrystal, assembly, growth method, electronic, magnetic, device

## Abstract

Cocrystal engineering is an advanced supramolecular strategy that has attracted a lot of research interest. Many studies on cocrystals in various application fields have been reported, with a particular focus on the optoelectronics field. However, few articles have combined and summarized the electronic and magnetic properties of cocrystals. In this review, we first introduce the growth methods that serve as the basis for realizing the different properties of cocrystals. Thereafter, we present an overview of cocrystal applications in electronic and magnetic fields. Some functional devices based on cocrystals are also introduced. We hope that this review will provide researchers with a more comprehensive understanding of the latest progress and prospects of cocrystals in electronic and magnetic fields.

**GRAPHICAL ABSTRACT:**
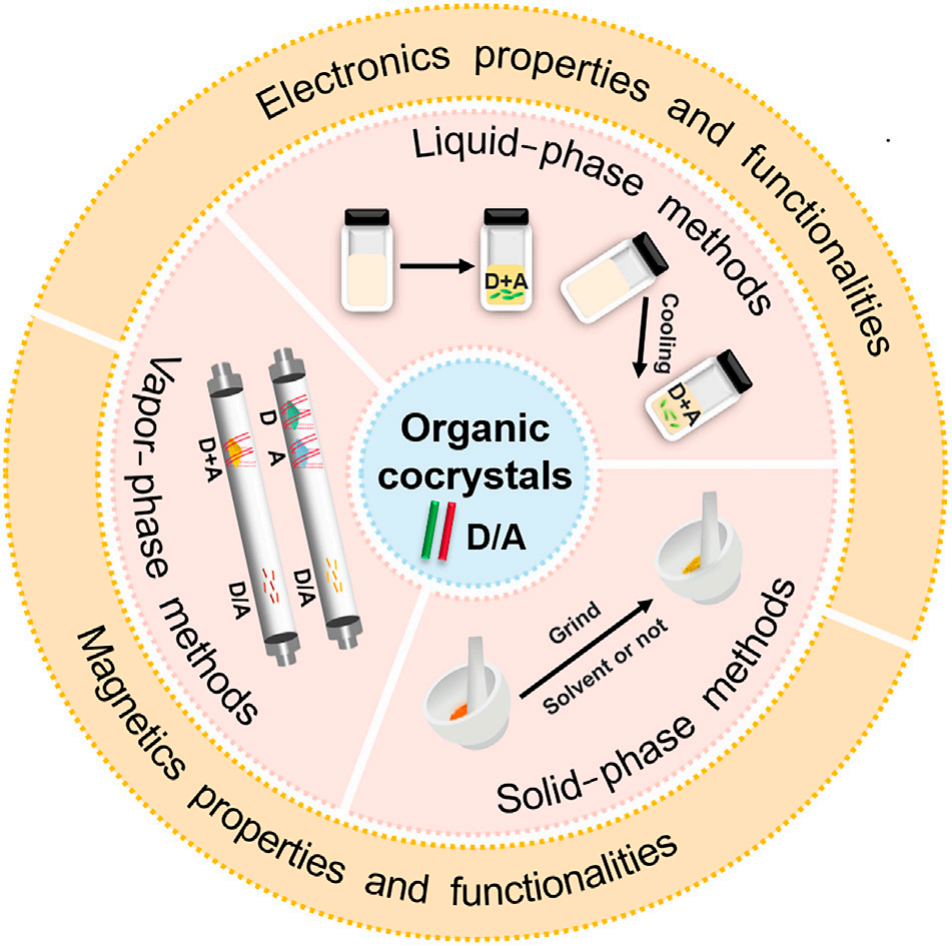


## Introduction

Organic semiconductor materials have outstanding characteristics, such as easy preparations, large-area solution processing, good flexibility, light weight, playing a crucial role in chemical engineering and materials design. To date, many advances have been made in the design and synthesis of high-performance organic crystals (Dong et al., 2013; Zhang X. et al., 2018; Yu P. et al., 2019; Qin et al., 2021). However, since these materials have a single component that only exhibits intrinsic properties, further applications are limited. Cocrystal engineering is a brilliant strategy that combines two or more components through noncovalent assembly (Yan and Evansa, 2014; Sun et al., 2020; Wang and Qin, 2021), which is promising in materials and chemistry science (Yan et al., 2011; Yan et al., 2012; Li et al., 2016; Lin et al., 2017; Zhou and Yan, 2019). The original constituent units display intrinsic properties, and more novel properties may emerge owing to the cooperativity effect between donor-acceptor (D-A) molecules (Li and Yan, 2018a; Sun et al., 2018; Huang et al., 2019; Zhou et al., 2020). For example, ambipolar charge-transport can be achieved by coassembling p-type and n-type semiconductors, which is difficult to realize for individual components (Goetz et al., 2016; Liu C. H. et al., 2019). Thus, organic cocrystal provides an effective way to construct multifunctional materials with desirable properties (Park et al., 2013; Liu et al., 2017; Wang Y. et al., 2018; Yu Y. et al., 2019).

Wöhler published the first report on cocrystals in 1844 (Wöhler 1844). After John Ferraris found the TTF-TCNQ (TTF, tetrathiafulvalene; TCNQ, 7,7,8,8-tetracyanoquinodimethane) cocrystal with high electrical conductivity in 1973 (Ferraris et al., 1973), people became increasingly interested in cocrystal engineering and conducted a wide range of correlational research (Sun et al., 2018). Especially in the electronic field, the charge transfer (CT) interaction and ambipolar transport gradually became the research hotspots. Following the discovery of the (BEDT-TTF)-F_2_TCNQ (BEDT-TTF, bis(ethylenedithiolo)tetrathiafulvalene; F_2_TCNQ, 2.5-difluorotetracyanoquinodimethane) cocrystal, which exhibits ambipolar CT behavior at low temperatures (Hasegawa et al., 2004), massive researches into cocrystals with high and balanced ambipolar charge-transport characteristics emerged (Park et al., 2013; Huang et al., 2019). Simultaneously, extensive studies on cocrystals with optoelectronic properties were carried out (Wang et al., 2016a). In 1995, the Kochi group proved that CT excitons generated in TCNB-based (TCNB, 1,2,4,5-tetracyanobenzene) cocrystals could relax into free carriers, implying that the cocrystals are ideal candidates for photoelectric conversion. Thereafter, many cocrystals with high carrier dissociation yields were synthesized (Hubig and Kochi, 1995). With the development of cocrystals, scientists went forward to a new field of magnetism and discovered that magnetic behavior was visible in CT cocrystals (Bolla et al., 2016). Since the discovery of the first all-organic multiferroic TTF-BA (BA, p-bromoaniline) in 2010 (Kagawa et al., 2010), lots of breakthroughs and developments in magnetic cocrystals have been made in the last 10 years (Wang and Zhang 2020).

This review systematically introduces the recent developments of cocrystals in electronic and magnetic areas because of their critical research value. The main preparation methods, involving the liquid-phase, vapor-phase, and solid-phase methods, are first introduced. Subsequently, achievements in these areas are elaborated from the following aspects: ambipolar transport, photoelectric conversion, magnetoelectric coupling, and magnetic anisotropy. Finally, the opportunities and challenges of cocrystal engineering in electronic and magnetic fields are proposed.

## Preparations of Organic Cocrystals

Currently, there are three main methods for effectively preparing cocrystals, including the liquid-phase, vapor-phase, and solid-phase methods (Braga et al., 2013; Hui and Christian, 2013; Huang et al., 2019). Since the growth methods significantly affect the properties of cocrystals, further affect the devices’ performances based on cocrystals, it is essential to select suitable growth conditions by considering the intrinsic properties of different components.

### Liquid-phase Methods

The liquid-phase methods are the most frequently used methods for preparing cocrystals owing to the advantages of low cost and easy preparation (Yan et al., 2014; Li and yan, 2018b; Lu et al., 2018). By adjusting some factors such as solvent type, temperature, and concentration, cocrystals of different morphologies and sizes can be obtained easily (Wang et al., 2021). Here, we mainly introduced three common liquid-phase methods: slow evaporation, drop-casting, and diffusion method.

**FIGURE 1:**
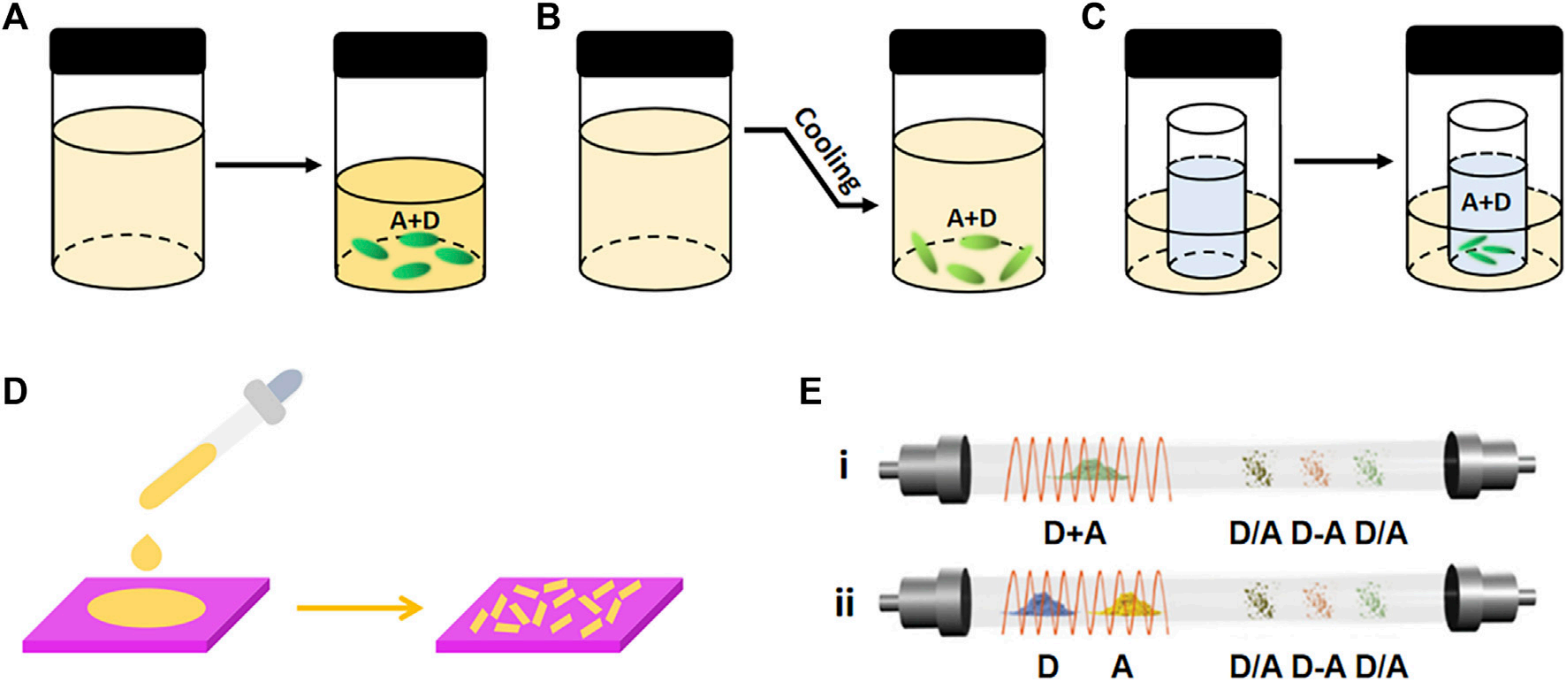
Schematic illustrations of the cocrystal growth processes of **(A)** slow evaporation, **(B)** slow cooling evaporation, **(C)** diffusion, **(D)** drop-casting, and **(E)** physical vapor transport (PVT) (Reproduced from Sun et al. (2019) with permission from WILEY-VCH, Copyright 2019.).

In the slow evaporation method, the mixture of donors and acceptors is dissolved in the organic solvent and then kept at room temperature (Figure 1A). As the solvent evaporates, raw components aggregate and crystallize as a result of the intermolecular interaction. The donors and acceptors should have similar and good solubility in the same solvent to avoid the precipitation of a single component (Sun et al., 2019; Wu et al., 2021). Since the solubility of raw components highly depends on the solvent type, the selection of solvent is very crucial. When changing the solvent type, the morphology and composition of cocrystal can be quite different. For instance, by using CH_2_Cl_2_ and tetrahydrofuran (THF) as the solvent, respectively, Wang et al. obtained a binary NDI-Cor (NDI, napthalenetetracarboxylic diimide; Cor, coronene) with ribbon structure and a ternary (NDI-Cor)·THF with block structure (Wang et al., 2020a). Slow cooling evaporation is based on the slow evaporation method, which is a method for growing cocrystals by controlling the temperature condition (Figure 1B). With this method, more components dissolve in the solution as the temperature increases, the raw materials crystallize as the temperature decreases. This method is more suitable for materials with moderate solubility at room temperature (Zhang et al., 2017a).

While the slow evaporation method is used to grow cocrystals with big sizes, the drop-casting method is used to prepare micro/nano cocrystals for constructing optoelectronic devices (Sun et al., 2018; Yan, 2015). By dropping an amount of solution on the prepared substrate, raw components gradually nucleate and crystallize with the volatilization of solvent in the droplet (Figure 1D). In this method, the solution concentration is a crucial factor affecting the micro-/nanostructures of cocrystals. Liu et al. revealed that the DMAQ (DMAQ, 4-(4-Dimethylaminostyryl)quinoline) and FDIB (FDIB, 1,4-diiodotetrafluorobenzene) with high concentration formed an M-DFC cocrystal with a two-dimension (2D) hexagonal microplate shape, whereas the low concentration formed a T-DFC cocrystal with 2D rhomboid-shaped microplate morphology (Liu Y. et al., 2019). Injecting a solution of raw materials into the nonvolatile solvent before drop-casting can induce cocrystals with unique morphologies. For example, after injecting a solution of pyrene and TCNB into an ethanol/water mixture, microtubes of pyrene-TCNB were collected on the quartz substrate (Sun et al., 2017).

The process of diffusion method is more complex, in which the raw materials are dissolved in a good solvent, and then a poor solvent (methanol, ether, or triethylamine) is diffused into the solution. The solubility of the solution gradually decreases as the poor solvent diffuses, and then the solution becomes saturated for crystallization (Figure 1C). The slow diffusion process guarantees the good quality and large size of cocrystal (Huang et al., 2019). Wang et al. assembled NDI-Δ with coronene (NDI-Δ, an organic naphthalenediimide-based triangle) by the diffusion method, obtained two bulk cocrystals of CNC-T and CNC-Q with good quality for the X-ray single-crystal structure characterization (Wang et al., 2020b).

### Vapor-phase Methods

Compared with the liquid phase methods, vapor phase methods are unrelated to materials’ solubility, which are suitable for materials with low solubility (Wang et al., 2021; Fang et al., 2017). The physical vapor transport (PVT) method is most popular (Figure 1E), using equipment consisting of a vacuum pump, a tubular furnace, a quartz tube, temperature controllers, and a gas path device. Under a flowing atmosphere of inert gas or in a vacuum, the original components in the high-temperature region sublimate and are subsequently transported to the low-temperature zone to form cocrystals. There are two types of PVT methods according to the sublimation points of the constituents (Figure 1E). The components are placed in the same sublimation region when the sublimation temperatures of the donors and acceptors are similar (Figure 1E-i). For example, two sizes of coronene-HAT(CN)_6_ (HAT(CN)_6_, 1,4,5,8,9,12-hexaazatriphenylene-hexacarbonitrile) were prepared by coevaporation in argon gas or vacuum (Liang et al., 2019). Another type of PVT method is appropriate for the constituents with significantly different sublimation points, in which the donors and acceptors are placed in two furnace regions (Figure 1E-ii). By placing the donors and acceptors in two furnace regions of 155°C and 190°C, respectively, the micro cocrystals of TMB-TCNQ (TMB, 3,3′,5,5′-tetramethylbenzidine) were obtained (Mezzadri et al., 2018). However, the products are difficult to separate, which is an inevitable problem when using this method to prepare cocrystals with different phases (Wang et al., 2021).

**FIGURE 2:**
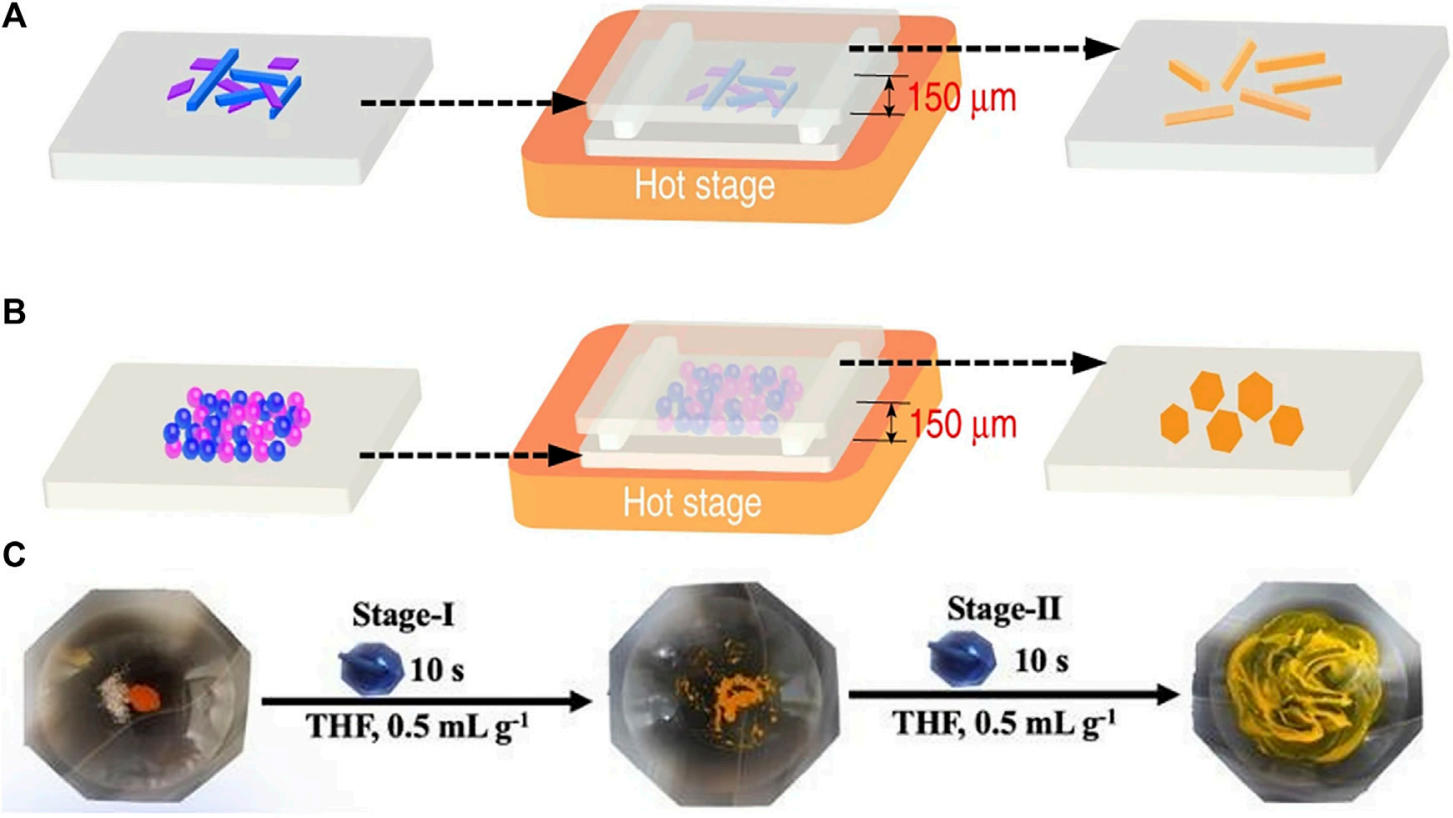
MAS apparatus for the growth of **(A)** one-dimension (1D) and **(B)** two-dimension (2D) fluoranthene-TCNB (Reproduced from Ye et al. (2019) with permission from Springer Nature, Copyright 2019.). **(C)** Liquid-assisted grinding procedures (LAG) for TC-OFN (TC, tetracene; OFN, octafluoronaphthalene) (Reproduced from Huang et al. (2020) with permission from American Chemical Society, Copyright 2020.).

The PVT method requires a vacuum environment and a long time, resulting in high cost and time-consuming (Sun et al., 2019). To solve this problem, Tao’s group proposed a microspacing in-air sublimation (MAS) method to grow a series of PAH-TCNB (PAH, polycyclic aromatic hydrocarbon) cocrystals, which exhibited (one-dimension) 1D needle-like (Figure 2A) or 2D plate-like morphologies (Figure 2B) (Ye et al., 2019).

### Solid-phase Methods

Solid-phase methods produce fewer organic cocrystals than the methods mentioned above. However, in recent years, these methods being commonly employed to prepare cocrystals due to the advantages of vacuum/heat-free conditions and a minimal amount of solvent or no solvent. The solid-phase methods can be divided into plain grinding and liquid-assisted grinding (LAG) methods. In the plain grinding method, raw materials are mixed according to a certain molar ratio in a mortar for grounding. This method is suitable for raw materials with poor solubility (Sun et al., 2018). As an example, in the grounding process, the yellow BQ and IP (BQ, p-benzoquinone; IP, 4-iodophenol) powders converted into red in several seconds, forming a 1:1 BQ-IP cocrystal (Carstens et al., 2020). Although the grinding method is fast and has a higher yield, the products always have small sizes and irregular morphologies. The other grinding method is liquid-assisted grinding (LAG). By adding a small amount of solvent during the grinding process, the interaction between donors and acceptors becomes stronger as the friction between the two substances increases, contributing to the cocrystallization of the components (Sun et al., 2018). Huang et al. successfully prepared TC-OFN (TC, tetracene; OFN, octafluoronaphthalene) by adding the THF solvent twice in a two-step LAG process (Figure 2C). This method produces cocrystals with better crystallinity and more controllable polymorphs (Huang et al., 2020).

## Electronic Properties and Functionalities

Since the discovery of highly conductive polyacetylene in 1977 (Chiang et al., 1977), people have been increasingly keen to explore the electronic properties of organic materials (Zhang et al., 2017b). In recent years, a large number of organic D-A complexes have been synthesized, which exhibit field-effect (Zheng et al., 2018; Mandal et al., 2020), photoresponse (Wu et al., 2014a), photovoltaic (Zhang et al., 2016), thermoelectric (Liang et al., 2020), and superconducting properties (Ferraris et al., 1973). With the development, the electronic properties of cocrystals may eventually be comparable to those of single crystals (Jiang et al., 2018). For example, p-type FETs based on DPTTA-DPNDI (DPTTA, meso-diphenyl tetrathia[22]annulene[2,1,2,1]; DPNDI, N, N′-bis(phenyl) naphthalene-1,4,5,8-bis(dicarbox-imide)) cocrystals exhibited a high transport property of 1.8 cm^2^ V^−1^ s^−1^, while the hole mobility of the pure DPTTA single crystals was only 0.7 cm^2^ V^−1^ s^−1^ (Zhang et al., 2014). The authors attributed the enhanced p-channel performance to the acceptor functioning as a good assistant in confining the stacking of donor molecules. Additionally, in 2012, the remarkable ambipolar semiconductor nature of mixed-stack cocrystals was predicted via density functional theory calculations, demonstrating that cocrystals have high potential in organic electronics, rivaling or even surpassing the best single-component organic crystals (Zhu et al., 2012). This review highlights the ambipolar transport and photoelectric conversion characteristics of organic cocrystals and their applications in organic field-effect transistors (OFETs) and photoresponse devices.

### Ambipolar Transport and OFET Devices

At present, researchers have made great progress in the synthesis of organic semiconductor materials with ambipolar properties (Zhang J. et al., 2018; Mandal et al., 2019a; Mandal et al., 2019b). Regardless, there are few high-performance and stable ambipolar materials in the ambient atmosphere because of the complexity and uncertainty of the synthesis route. It is inspiring that the cocrystal strategy can effectively integrate donors and acceptors into a single crystal system to obtain hole or electron carriers channels (Sun et al., 2019). This “molecular level heterojunction” provides an alternative approach to realize ambipolar transport through an easy-to-process method of low cost and high efficiency. Therefore, the cocrystals are considered promising active elements to construct ambipolar OFETs with high performance. Herein, we introduce the latest achievements in OFETs based on cocrystals and discuss the influencing factors on the adjustable ambipolar properties, including energy level, molecular stacking pattern, and molecule structure, from theoretical and experimental perspectives.

**FIGURE 3:**
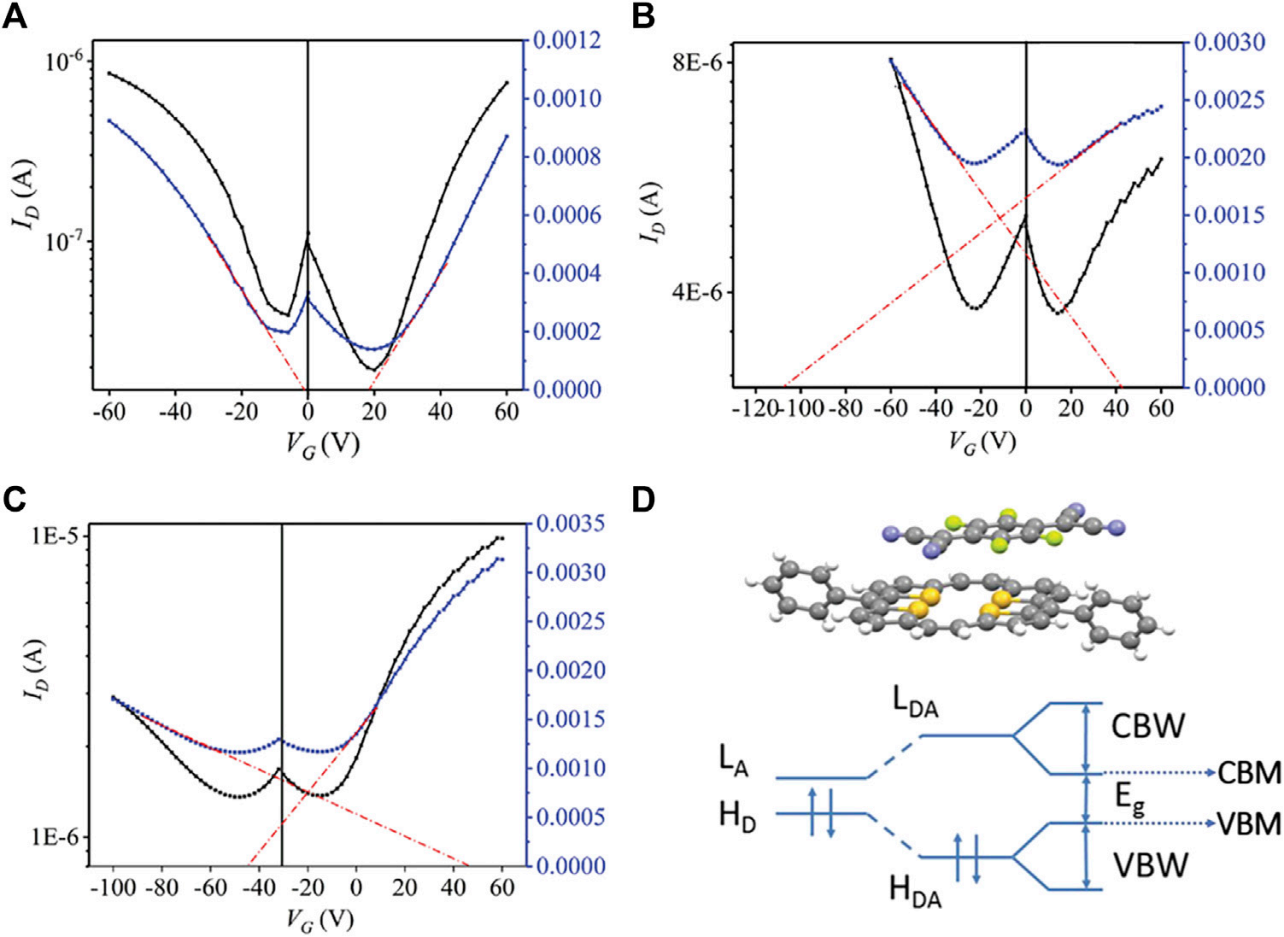
Transfer characteristics of OFETs based on **(A)** DPTTA-F_1_TCNQ, **(B)** DPTTA-F_2_TCNQ, and **(C)** DPTTA-F_4_TCNQ. **(C)** Schematic drawing of the band structure of DPTTA-F_X_TCNQ (L_A_ and H_D_ refer to the lowest unoccupied molecular orbitals (LUMOs) of isolated acceptors and highest occupied molecular orbitals (HOMOs) of donors, L_DA_ and H_DA_ refer to LUMOs and HOMOs in DPTTA-F_X_TCNQ, CBW and VBW refer to conducting bandwidth and valance bandwidth, CBM and VBM refer to conducting band minimum and valance band maximum.) (Reproduced from Liang et al. (2020) with permission from WILEY-VCH, Copyright 2019.).

Compared with single-component materials, the electronic properties of cocrystals can be easily regulated by altering the donors or acceptors (Wang et al., 2021; Sun et al., 2018). Using molecules with increasing F atoms as acceptors is a typical method for regulating the charge transport properties of cocrystals (Liu H. et al., 2019; Wei et al., 2020). The increasing electron affinity of acceptors usually results in enhanced CT degree, which has a significant impact on the molecular stacking pattern and the energy levels, further influencing the charge transport properties of cocrystals. In comparison to DPTTA-TCNQ that had no CT between D-A molecules, DPTTA-F_x_TCNQ (FxTCNQ, fluorinated derivatives of 7,7,8,8,-tetracyanoquinodimethane, X = 2, 4) exhibited enhanced CT features with almost identical overlap patterns between D-A molecules along the stacking direction (Liang et al., 2020). Furthermore, Liang et al. proved that the CT degree of DPTTA-F_x_TCNQ (X = 1, 2, 4) increased as F atoms of the acceptor molecules increased. The calculated transfer integrals displayed an increasing tendency, indicating that the electronic coupling improved from DPTTA-F_1_TCNQ, DPTTA-F_2_TCNQ to DPTTA-F_4_TCNQ. The relatively strong intermolecular electronic couplings led to more dispersed valence bands and conducting bands, as well as narrower band gaps (Zheng et al., 2015). OFETs based on these cocrystals all exhibited ambipolar transport characters. The mobilities were 0.15 cm^2^ V^−1^ s^−1^ (μh) and 0.24 cm^2^ V^−1^ s^−1^ (μe) for DPTTA-F_1_TCNQ, respectively; 1.01 cm^2^ V^−1^ s^−1^ (μh), 0.27 cm^2^ V^−1^ s^−1^ (μe) for DPTTA-F_2_TCNQ; and 0.11 cm^2^ V^−1^ s^−1^ (μh), 0.46 cm^2^ V^−1^ s^−1^ (μe) for DPTTA-F_4_TCNQ. It should be noted that the predominant carrier in DPTTA-F_4_TCNQ were electrons, while that in DPTTA-F_1_TCNQ were holes (Figures 3A–C). The n-doping in the DPTTA-F_4_TCNQ was contributed to the deepest conducting band minimum (CBM) level caused by the strongest electron affinity of F_4_TCNQ. On the contrary, the F_1_TCNQ complex preferred to be p-type doped because of the highest valence band maximum (VBM) level (Figure 3D) (Liang et al., 2020). This study shed light on the design of cocrystals with ambipolar transport behaviors.

**FIGURE 4:**
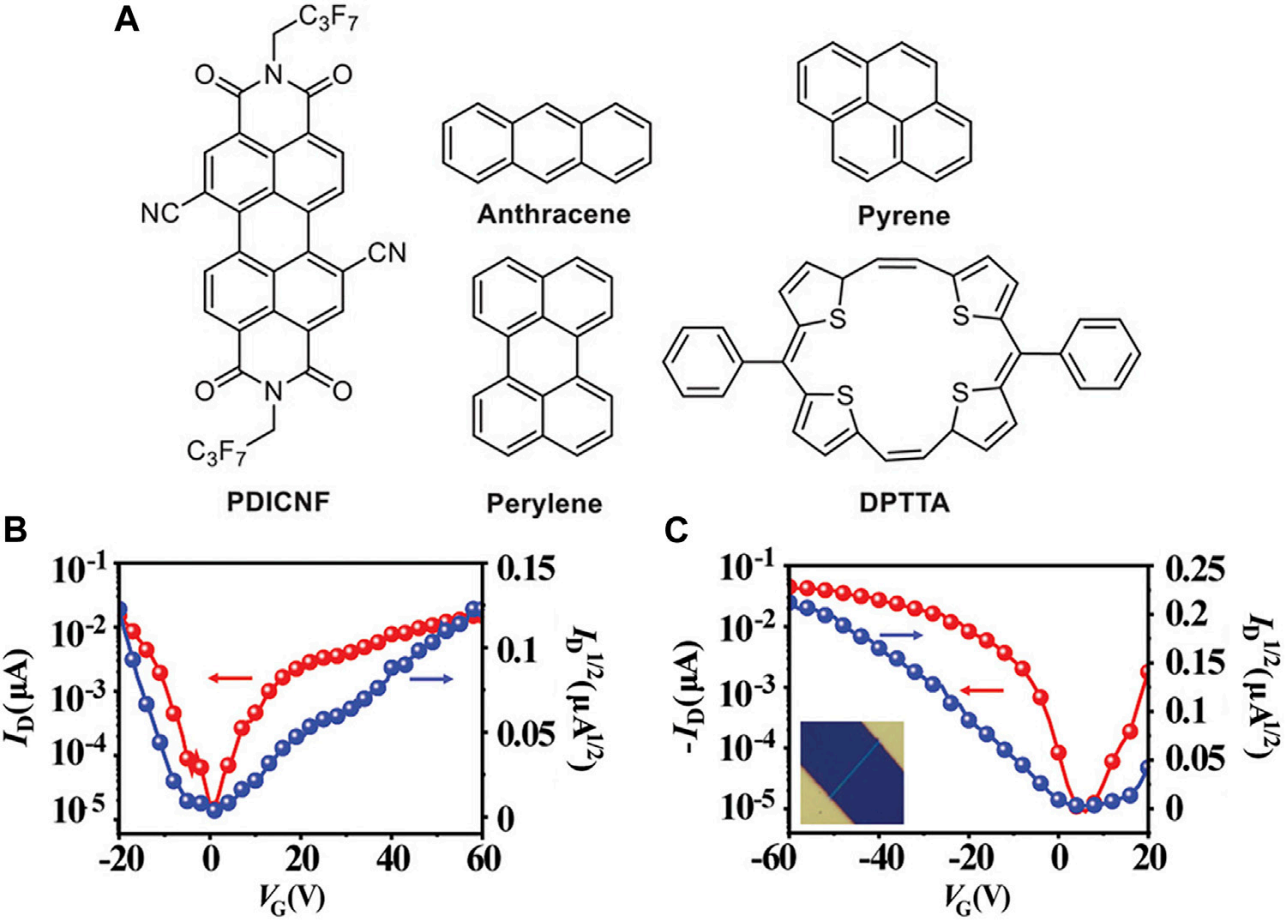
Chemical structures of **(A)** anthracene-PDICNF, pyrene-PDICNF, and perylene-PDICNF. The **(B)** n-type and **(C)** p-type transfer characteristics of DPTTA-PDICNF (Reproduced from Yu et al. (2021) with permission from Wiley-VCH, Copyright 2021.).

In addition, Yu et al. also achieved the ambipolar charge transport in cocrystals by assembling acceptors with donors of different aromatic conjugated backbones. With the aromatic conjugated backbone of donors increased, the energy levels of supramolecular hybrid orbitals in D/A pairs were higher, contributing to the CT interaction (Zhang X. et al., 2018; Dasari et al., 2019). They synthesized four cocrystals using PDICNFN (PDICNF, N′-bis(perfluorobutyl)-1,7- dicyanoperylene-3,4:9,10-bis (dicarboximide) as the acceptor and anthracene, pyrene, perylene, and DPTTA as the donors (Figure 4A). The theoretical calculation of density functional theory (Jiang et al., 2018) suggested that in the range of -3.82 eV to -4.07 eV, the cocrystals maintained similar lowest unoccupied molecular orbitals (LUMOs), slightly higher than PDICNF. The highest occupied molecular orbitals (HOMOs) of the cocrystals increased from −5.75 eV (anthracene-PDICNF) to −4.84 eV (DPTTA-PDICNF), higher than the corresponding donors. Meanwhile, the extended π-conjugated system of the donor molecule DPTTA further promoted electronic coupling. Therefore, the DPTTA-PDICNF was hypothesized to have better charge transport properties, which were confirmed by well-balanced field-effect mobilities of 2.0 × 10^–2^ cm^2^ V^−1^ s^−1^ for the holes and 1.7 × 10^–2^ cm^2^ V^−1^ s^−1^ for the electrons (Figures 4B,C). The anthracene-PDICNF, pyrene-PDICNF, and perylene-PDICNF only showed n-transport properties. Notably, pyrene-PDICNF had carrier mobility of 0.19 cm^2^ V^−1^ s^−1^, the highest value ever found in PDI-based cocrystals (Yu et al., 2021). This research also provided a guide for synthesizing cocrystals with ambipolar transport properties.

**FIGURE 5:**
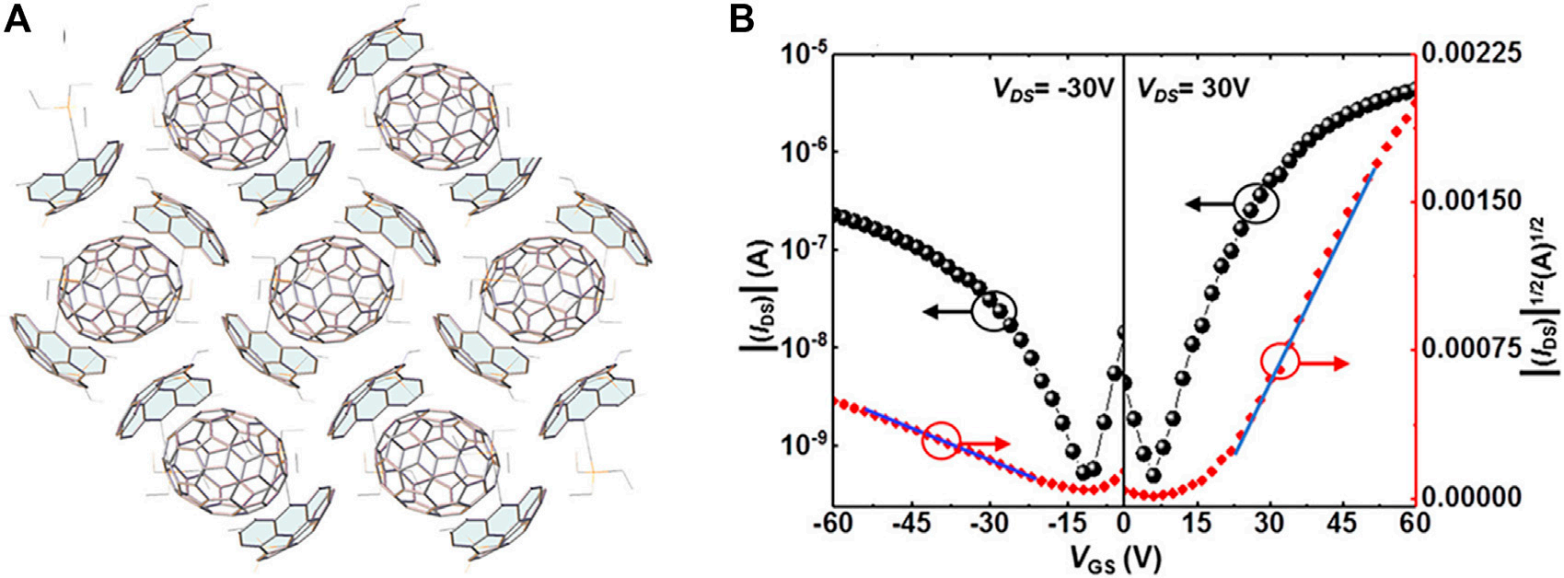
**(A)** Crystal structure and **(B)** transfer characteristics of 1-C_70_ (Reproduced from Gao et al. (2020) with permission from American Chemical Society, Copyright 2020.).

Considering that the electrical properties of cocrystals highly rely on the molecular structures (Zhu et al., 2014; Ai et al., 2017), selecting D-A molecules with matching structures as constituents is another strategy to achieve the ambipolar properties. For example, DTTCNQ [DTTCNQ, 4,8-bis(dicyanomethylene)-4,8-dihydrobenzo(1,2-b:4,5-b')-dithiophene] with the extended π-conjugated system may better match the donor molecule than TCNQ. The increasing conjugated system and partial charge-transfer character in DPTTA-DTTCNQ enhanced D-A interactions by shortening the D-A distance and formed a quasi-2D ambipolar transport network. There were both superexchange and indirect paths for charge transport. Thus, high charge-transport properties could be expected by extending the π-conjugated systems despite the weak electron-accepting ability of DTTCNQ (Qin et al., 2014). In addition to applying the similar structures of D-A molecules, complementary geometry also facilitates charge transport. Recently, Gao et al. synthesized diindeno (4,3,2,1-fgh i:4′,3′,2′, 1′-Opqr) perylene, which was a subunit of C_70_. This buckybowl skeleton was functionalized at the meta-positions with triethylsilyl-ethynyl (TES-ethynyl) (1), ensuring the solubility and stability of the buckybowl skeleton and forming 1D concave-in-convex stacking columns with a hole mobility of 0.31 cm^2^ V^−1^ s^−1^. Considering the potential shape complementarity, one was blended with the C_70_ acceptor to obtain a novel cocrystal. The TES-ethynyl helped form buckybowls arrangement with strong concave-convex interactions. As shown in Figure 5A, each C_70_ molecule made contact with six bowl molecules, forming 2D cocrystals and facilitating the effective transmission of charge carriers through curved surfaces. The OFET measurements demonstrated that the cocrystal possessed ambipolar property, with electron and hole mobilities of 0.40 cm^2^ V^−1^ s^−1^ and 0.07 cm^2^ V^−1^ s^−1^, respectively (Figure 5B) (Gao et al., 2020), indicating that the complementary structures were promising for the ambipolar transport of cocrystals.

All in all, cocrystal engineering provides a practical and simple strategy for systematically controlling the operation mode (ambipolar, or p-/n-type) of the transistor by modifying the components. Through co-crystallization, the band gaps of the semiconductors can be adjusted to facilitate the energy matching between the cocrystal Frontier orbitals and the work function of the injected electrodes, which is beneficial to efficient charge injection to improve the OFETs performance.

### Photoelectric Conversion and Photoresponse Devices

Photoresponse materials play an important role in the organic optoelectronics field, which can transfer optical signals into electrical signals, have wide applications in photodetectors (Altaqui et al., 2021), photoswitches (Kellner and Berlin, 2020), phototransistors (Gelinck et al., 2010; Dong et al., 2012), and optical imaging (Chen et al., 2021). An idea photoresponse device should ensure the processes of photon absorption, exciton dissociation, and charge carrier transport (Najafov et al., 2010; Ostroverkhova, 2016). The features of modulating absorption, special D-A molecular interfaces engender cocrystals serving as outstanding candidates for photoresponse (Wu et al., 2014b; Wang et al., 2016b; Wang et al., 2020d; Singha et al., 2021). In this section, besides the superiorities, we will discuss the structure-property relationship of cocrystals in photoresponse and introduce recent high-performance photoresponse devices based on micro/nano cocrystals.

**FIGURE 6:**
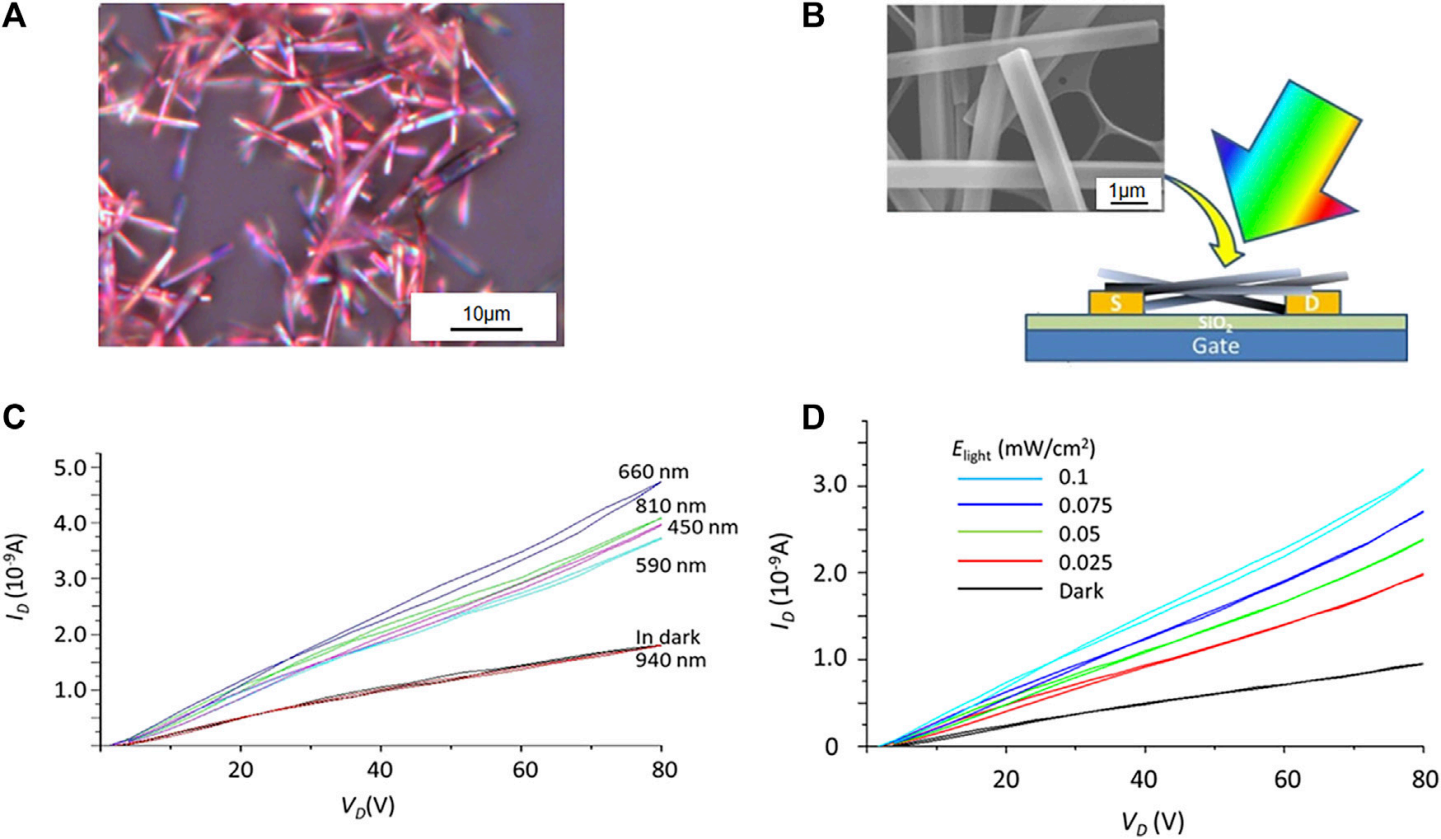
**(A)** Optical microscopy image and **(B)** schematic diagram of OFET device of C_60_/3,5-TPP, inset: scanning electron microscopy (SEM) image of C_60_/3,5-TPP. **(C)** Wavelength dependence of the output characteristics of a C_60_/3,5-TPP phototransistor at V_G_ = 80 V when illuminated with different LEDs (E_ligh_t = 0.1 mW/cm^2^). **(D)** Output characteristic curves of the phototransistor based on C_60_/3,5-TPP at V_G_ = 80 V when illuminated with 660 nm light of different intensities (E_light_) (Reproduced from Wakahara et al. (2020) with permission from American Chemical Society, Copyright 2020.).

In CT cocrystals, a new CT state generates between donors and receptors because of the intermolecular interaction, allowing for redshift absorption (Siegmund et al., 2017). When strong CT interaction occurs, the CT absorption band moves to the long-wavelength region (Sun et al., 2019; Tang et al., 2021). By virtue of this phenomenon, photoresponse in the infrared or near-infrared region can be achieved. Wakahara et al. fabricated an OFET, in which 3,5-TPP/C_60_ [3,5-TPP, 5,10,15,20-tetrakis(3,5-dimethoxyphenyl)porphyrin] served as the semiconductor layer (Figures 6A,B). The four dimethoxyphenyl substitutions endowed the 3,5-TPP with a strong electron-donating ability that enhanced the CT interaction with C_60_. A new CT absorption band emerged at 600–800 nm. The channel current (ID) increased as the light intensity (Elight) increased when light-emitting diodes with emission peaks in the visible-to-NIR region (450, 590, 660, 810, and 940 nm) were used to illuminate the OFET (Figure 6C). The increasing current at 810 nm was attributed to the CT state in the C_60_/3,5-TPP cocrystals were excited to generate excitons that subsequently separated. Due to the CT absorption band and component bands, the phototransistor based on the C_60_/3,5-TPP cocrystal exhibited a strong photoresponse at 660 nm, and the measured photosensitivity was 4.5 (0.05 mW/cm^2^) (Figure 6D) (Wakahara et al., 2020).

In addition to the modulating absorption, the plenty of D-A interfaces in cocrystals ensure efficient exciton dissociation, contributing to the photoelectric conversion (Wang et al., 2017; Sun et al., 2019). The CT excitons in cocrystals are considered a highly localized excitation-pair state and then relax to the ground state or dissociate into free carriers (Hubig and Kochi, 1995; Sun et al., 2019). Meanwhile, the hybrid molecular orbital (MOs) at D-A interfaces hinder the reversed charge-transfer process, which prevents the exciton recombination, ultimately affects the photoresponse. Zhang’s group selected TMIQ (TMIQ, 8,8,18,18-tetramethyl-8,18-dihydroindolo(1,2,3-fg)indolo(3′,2′,1′:8,1)quinolino[2, 3-b]acridine) as the donor and synthesized it with acceptors of CA, FA, and TCNQ (CA, p-chloranil; FA, p-fluoranil). Under photoexcitation, the charge was redistributed between D-A molecules, which enhanced the density of charge carrier and thus induced the photocurrent. The large energy barriers in TMIQ-CA and TMIQ-FA were 0.4 and o.96 eV, which hindered the reversed charge-transfer processes, while the energy barrier was lost in TMIQ-TCNQ. Therefore, only the TMIQ-CA and TMIQ-FA exhibited photoresponse properties. However, the result appeared that the TMIQ-CA showed the best photoresponse despite having a smaller energy barrier than TMIQ-FA. It may be attributed to that the CH … C bonds network of donors in TMIQ-CA further promoted the excitons separation and carrier transport. Under ultraviolet (UV) illumination, the phototransistor based on TMIQ-CA had a maximum photocurrent on/off ratio of 353, photoresponsivity of 3.0 × 10^3^ A W^−1^, detectivity of 1.4 × 10^14^ Jones, and external quantum efficiency of 2.4 × 10^6^%, which were the best values among all reported organic cocrystals (Wang et al., 2020d).

**FIGURE 7:**
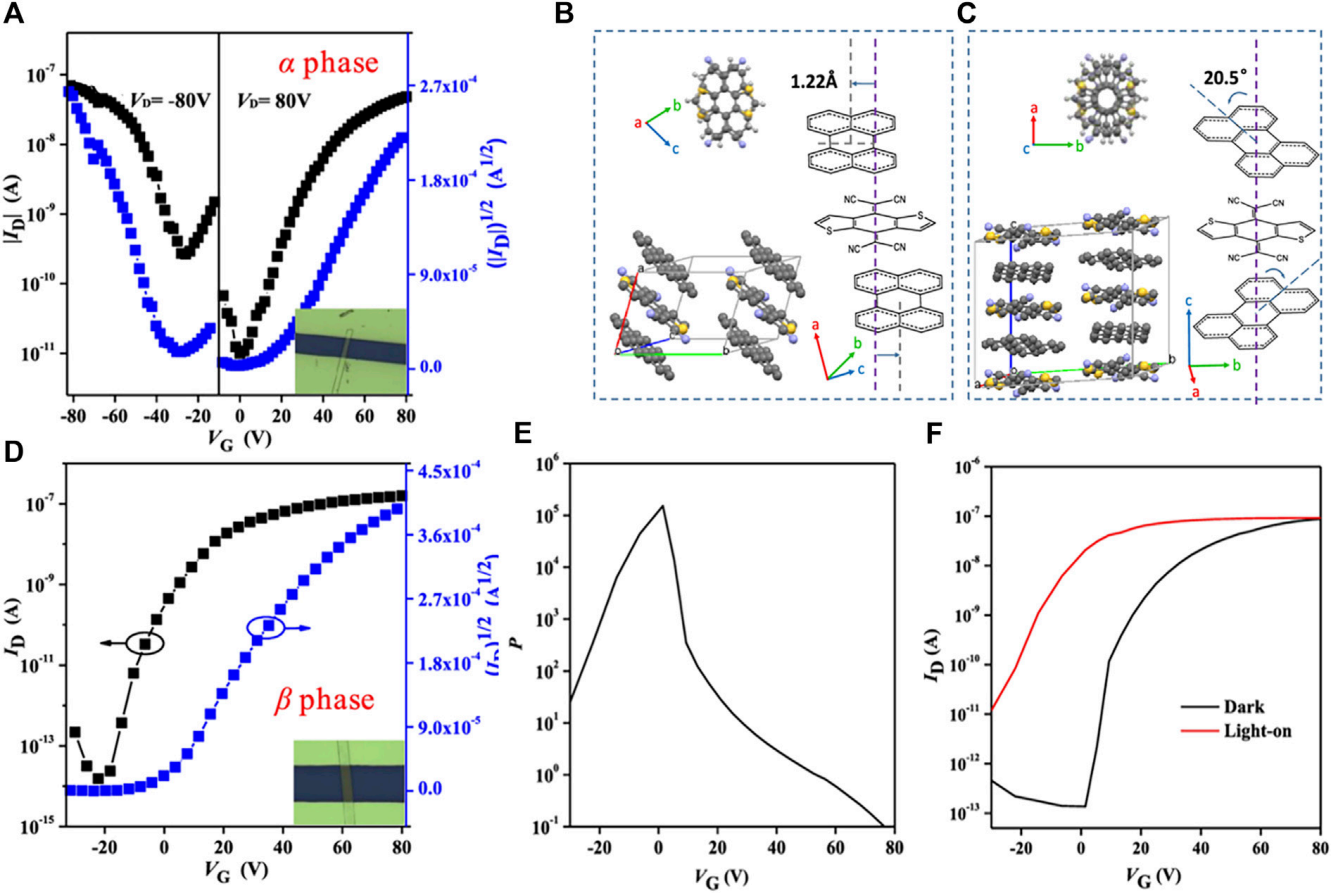
**(A)** Transfer characteristics of the OFET based on α-phase perylene-DTTCNQ. Crystal packing structures of **(B)** α-phase perylene-DTTCNQ and **(C)** β-phase perylene-DTTCNQ. **(D)** Transfer characteristics of the OFET based on α-phase perylene-DTTCNQ. **(E)** Photoresponse characteristics of the β-phase measured in the dark and under illumination with a light intensity of 274.2 mW cm^−2^ (V_DS_ = 80 V). **(F)** Photosensitivity of the β-phase OFETs under irradiation at different gate voltages (Reproduced from Jin et al. (2020) with permission from American Chemical Society, Copyright 2020.).

It is worth noting that the optoelectronic properties of cocrystals are also closely related to the molecular stacking structure (Park et al., 2013; Zhang et al., 2017b). Cocrystals with identical components but different stacking structures exhibit different photoresponse properties (Goetz et al., 2016). It was proposed that the (perylene)_1_-TCNQ with segregated-stacking mode had better photoresponse properties than the (perylene)_3_-TCNQ with mixed-stacking mode, which was unfavorable for the exciton dissociation (Zhu et al., 2015). A recent study reported that the cocrystals with different phases also showed different photoresponse properties. Jin et al. synthesized α-phase and β-phase cocrystals composed of perylene and DTTCNQ through homogeneous and heterogeneous nucleation, respectively. Thereinto, the α-phase cocrystal exhibited ambipolar transporting, but the semiconducting feature and photoresponse were low (Figure 7A). Compared to the brick-type α-cocrystal, β-cocrystal had a 20.5° rotation angle between D-A molecules, more like a columniform type (Figures 7B,C). This packing mode avoided steric hindrance but caused the vanish of the p-type channel (Figure 7D). Nonetheless, the photocurrent of the device based on β-cocrystal increased sharply under the illumination. The photosensitivity reached 1.5 × 10^5^ at V_G_ of 1 V, and the photoresponsivity was 28.2 mA W^−1^ (Figures 7E,F) (Jin et al., 2020).

**FIGURE 8:**
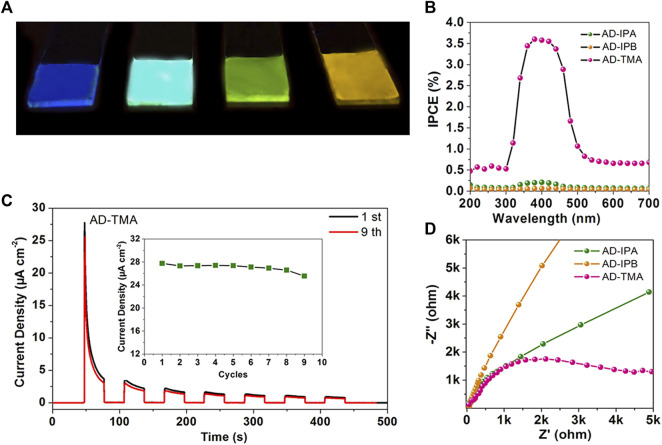
**(A)** Photographs of AD, AD-IPA, AD-IPB, and AD-TMA films under UV light. **(B)** Transient current density-time characteristic of the AD-TMA thin film for reusable tests without bias potential, inset: the maximal current density of the first on-off cycle measured at different reusable tests. **(C)** Incident photon-to-current efficiency of the three cocrystal thin films. **(D)** Electrochemical impedance spectroscopy Nyquist plots of three cocrystal thin films (the bias is −0.5 V) (Reproduced from Yang et al. (2020) with permission from American Chemical Society, Copyright 2020.).

Thanks to the advantages in photoelectric conversion, cocrystals have been widely used in photoresponse. Nowadays, novel ways for synthesizing cocrystals with photoresponse properties are being developed (Dong et al., 2012; Wang C. et al., 2018). For instance, molecule-level heterojunction cocrystal thin films, which promote the migration and separation of excitons, display great potential in achieving photoresponse. Yang et al. assembled AD with IPA, IPB, and TMA (AD, acridine; IPA, isophthalic acid; IPB, 5-bromoisophthalic acid; TMA, trimesic acid) to obtain three cocrystal thin films of AD-IPA, AD-IPB, and AD-TMA (Figure 8A). Among the three cocrystal thin films, the AD-TMA thin film exhibited the best photoresponse. The high crystallinity of the AD-TMA thin film benefited the transfer of charge carriers. Besides, the TMA anions layer and AD cation layer formed an internal electric field that promoted the efficient charge carriers separation. In a three-electrode system, the photocurrent density of the AD-TMA thin film electrode rapidly increased to 27.79 μA/cm^2^ (I_light_) under the on-off cycle’s illumination (30 s). After switching off the irradiation, the low photocurrent density is 0.002 μA/cm^2^ (I_dark_) (Figure 8B). The maximum current on/off ratio of the AD-TMA cocrystal thin film was 13,895 (I_light_/I_dark_), much higher than that of carbon nitride nanotube membranes, metal-organic framework materials in electrolytes, and the optoelectronic devices composed of inorganic perovskite and organic single crystal, indicating the exceptional sensitivity to light. Furthermore, the incident photon-to-current efficiency of the AD-TMA thin film was highest (Figure 8C). The fast CT rate was also confirmed by the lowest CT resistance (Figure 8D) (Yang et al., 2020). Recently, Wang et al. successfully fabricated a vertical photodetector device based on the 2D cocrystal film of ZnTPP (ZnTPP, 5,10,15,20-tetraphenyl-21H,23H-porphine Zinc) and C_60_. The photoresponsivity of this large-area cocrystal film was as high as 2,424 mAW^−1^ at 800 nm, combined with fast response times and high external quantum efficiency of 376%, further proving the superiority of cocrystal film in photoresponse (Wang et al., 2020c).

The cocrystal strategy provides a fascinating avenue for constructing materials with photoresponse by rationally selecting the donors and acceptors. The features of strong intramolecular interaction and unique structure facilitate an efficient photoelectric conversion. Nevertheless, the ultimate goal is to achieve more cocrystals with high-performance photoresponse, which requires further exploration and expansion of the co-crystalline system.

## Magnetic Properties and Functionalities

Organic magnetic materials are applied in sensors (Xu et al., 2017), magnetic recording (Zhang et al., 2008; Wei et al., 2019), microwave devices (Ustinov et al., 2007), magnetic memories (Bibes and Barthélémy, 2008; Vopson, 2016), and gyrators (Zhai et al., 2009) have aroused great interest in recent years. Cocrystal engineering synthesizes two or more components, has emerged as an intelligent way to design and tailor the multifunctional magnetic properties of organic materials (Hu and Zhang, 2020; Wang et al., 2021). Although research on cocrystals in magnetic field began later, it is gaining prominence (Wang and Zhang, 2020). This section mainly introduces the magnetic properties of cocrystals and then focuses on the multiferroic cocrystals. Potential applications in magnetic-field sensors and magnetic memory devices are also proposed.

**FIGURE 9:**
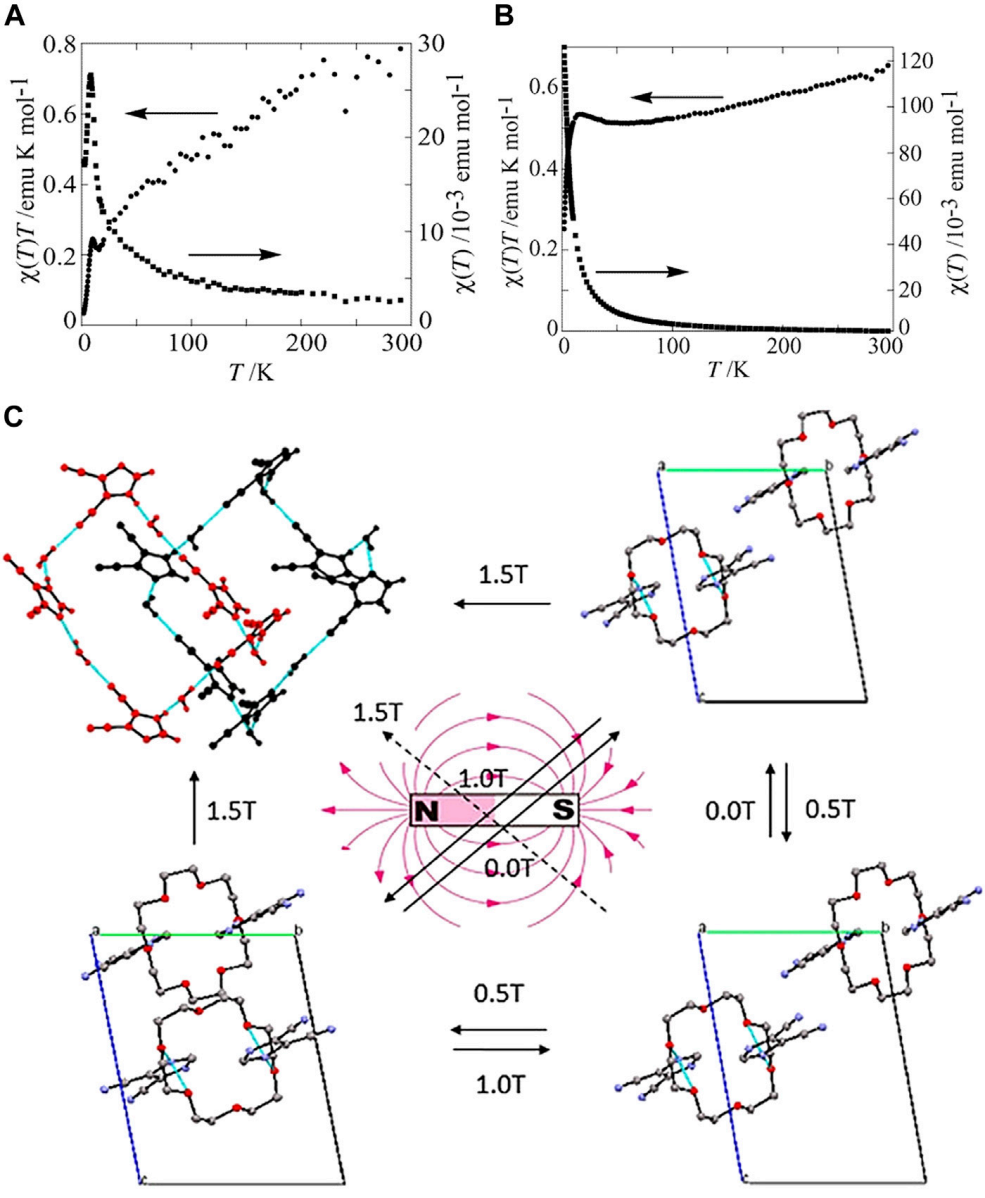
**(A)** Temperature dependence of the static susceptibility x and xT of (HMTTF)-[Ni(mnt)_2_]. **(B)** Temperature dependence of static x and xT for (ChSTF)-[Ni(mnt)_2_]. (Reproduced from Nakajima et al. (2004) with permission from American Chemical Society, Copyright 2004.). **(C)** Transformations of 18-Crown-6/4,5-dicyanoimidazole and 18-Crown-6/1,2,4-triazole under an external magnetic field with various strengths (Reproduced from Luo et al. (2017) with permission from American Chemical Society, Copyright 2017.).

Recently, many works toward synthesizing cocrystals with magnetic properties have been reported (Xu et al., 2016a; Zenno et al., 2021). It is proposed that the magnetic properties of cocrystals highly depend on the staking modes of D-A molecules (Yuan et al., 2018). According to the previous studies, the materials with separate-stacking mode usually exhibit ferromagnetism, while those with mixed-stacking mode often exhibit antiferromagnetism. For example, (EDO-TTFI_2_) [M(mnt)_2_] (EDO-TTFI_2_, diiodoethylenedioxy-TTF; mnt, maleonitrile dithiolate; M = Ni, Pt) with segregated columns showed ferromagnetic properties, while (BMDT-TTF)_2_[M(m-nt)_2_] (BMDT-TTF, bis(methylenedithio-TTF) with a mixed-stacking structure was an antiferromagnetic model (Nishijo et al., 2000; Torrent et al., 2002). Takehiko Mori et al. prepared CT (charge transfer) complexes of (HMTTF)-[Ni(mnt)_2_] and (ChSTF)-[Ni(mnt)_2_] (HMTTF, bis(trimethylene)-tetrathiafulvalene; ChSTF, 2,3-cyclohexylenedithio-1,4-dithia-5,8- diselanafulvalene), which had mixed-stacking modes, both exhibited antiferromagnetism. The xT minima of (HMTTF)-[Ni(mnt)_2_] and (ChSTF)-[Ni(mnt)_2_] showed around 16 and 55 K, respectively, while the xT peaks formed at 8 and 16 K (Figures 9A,B). The disappeared ESR signal at low temperature further demonstrated the antiferromagnetic transition of two CT complexes. However, the ferromagnetic anomaly of the (HMTTF)-[Ni(mnt)_2_] was discovered owing to the different g values of the donor and the anion or the ferromagnetic interaction of the [Ni(mnt)_2_] anions (Nakajima et al., 2004). Another work realized the reversibly stretching of cocrystals by applying a magnetic field with various strengths. The distance between molecules in 18-Crown-6/4,5-dicyanoimidazole was stretched under a magnetic field of 0.5 or 1 T strength. In comparison, the magnetic field of 0.5 T strength could alter the stretching distance of molecules in 18-Crown-6/1,2,4-triazole (Figure 9C). In consequence, the physical/chemical properties of the two cocrystals were altered (Luo et al., 2017). Ultimately, the two cocrystals were separated completely under the magnetic fields of 1.5 and 1.0 T strengths, respectively. These works promoted the development of functional organic cocrystals in the magnetic field.

**FIGURE 10:**
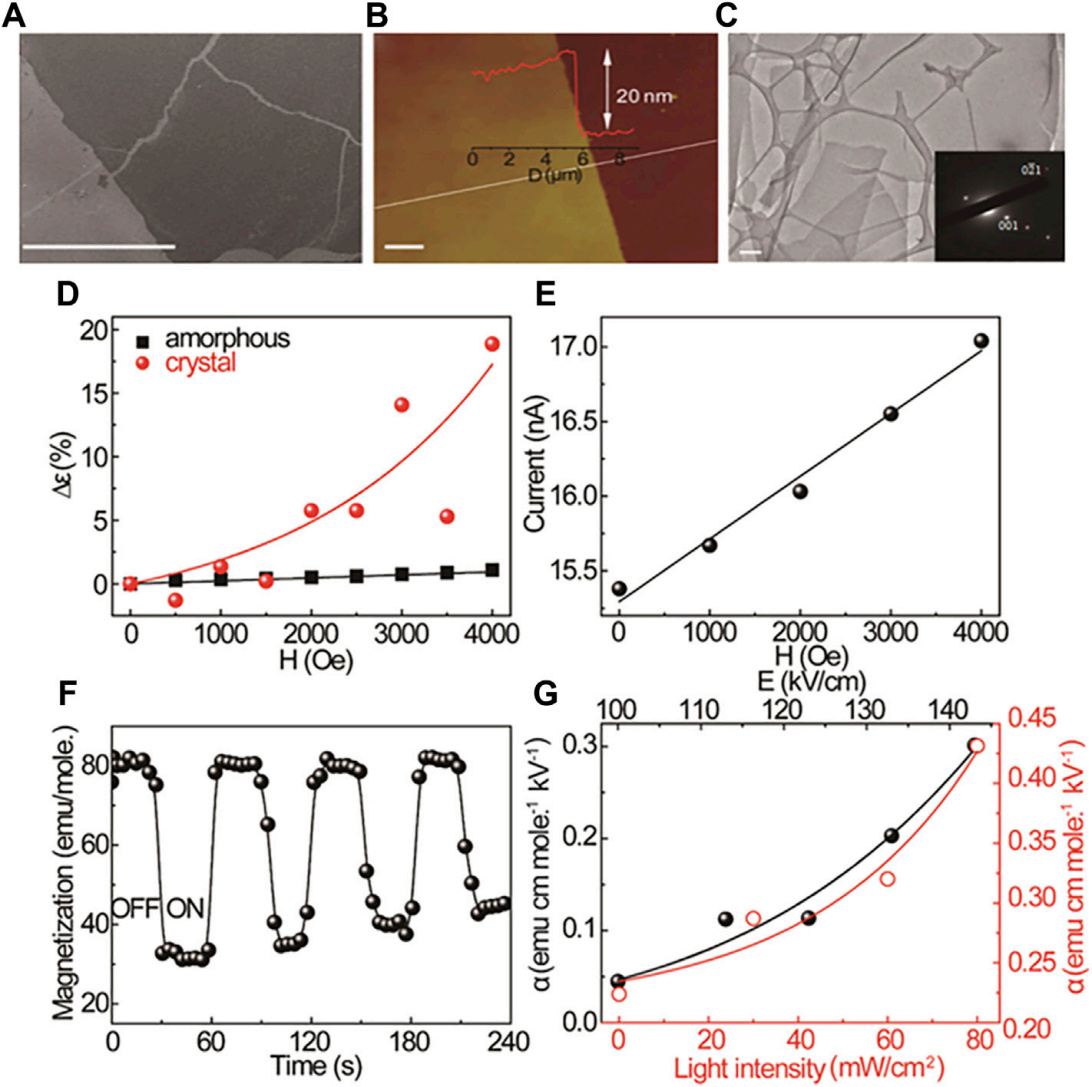
**(A)** Scanning electron microscopy (SEM), **(B)** atomic force microscopy (AFM), and **(C)** transmission electron microscopy (TEM) images of 2D TTF-C_60_ film, inset: SAED pattern. **(D)** Magnetic-field-dependent dielectric constant of amorphous and crystallized 2D TTF-C_60_ films. **(E)** Magnetic-field-dependent photocurrent (at 0.2 V) of crystallized 2D TTF-C_60_ film; **(F)** The tuning of magnetization of a crystallized 2D TTF-C_60_ films by switching an electric field on and off. **(G)** Electric-field-dependent and lightintensity-dependent magnetoelectric coupling coefficient of crystallized 2D TTF-C_60_ films (Reproduced from Xu et al. (2019) with permission from American Chemical Society, Copyright 2019.).

With the development of magnetic cocrystals, the multiferroic properties of cocrystals are taken seriously (Naka and Ishihara, 2016; Xu et al., 2016b; Qin et al., 2015a). In contrast to the materials with single magnetic properties, the multiferroic materials simultaneously exhibit two or more iron characteristics, including ferromagnetism, ferroelectricity, and ferroelastricity (Wang et al., 2021). Significantly, the materials with ferromagnetism and ferroelectricity can induce a magnetoelectric coupling effect that has prompted great concern. In this regard, the supramolecular structure of D-A-D-A … and the exchange interactions in CT cocrystals allow for ordered and controllable coupling of the electric and magnetic interactions (Wang and Zhang, 2020). Xu et al. assembled TTF with C_60_ to form a 2D cocrystal film (Figures 10A–C). In the 2D TTF-C_60_ films, the external magnetic field induced the conversion from singlet CT state to triplet CT state. More dipoles generated with triplet exciton density enhancement, and the ultimate polarization indicated the magnetoelectric coupling. The TTF-C_60_ films exhibited a magnetic-field-controlled magnetodielectric effect (Figure 10D). And the magnetoconductance further suggested the magnetoelectric coupling. With the magnetic field increased, the triplet CT state in the 2D TTF-C_60_ films enhanced the interaction of excitons and polarons, more triplet excitons dissociated into charge carriers, finally, the current increased (Figure 10E). Moreover, as the electric field and photoexcitation enhanced the magnetization of TTF-C_60_ films, the magnetoelectric coupling could be strengthened (Figures 10F,G) (Xu et al., 2019). Overall, magnetoelectric coupling control in 2D TTF-C_60_ films was realized, and their magnetic-field-dependent photoresponse property could be applied in magnetic-field sensors.

**FIGURE 11:**
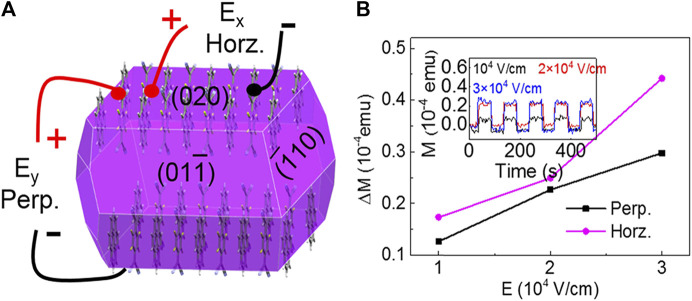
**(A)** The predicted crystal morphology and the schematic of applying horizontal (E_x_) and perpendicular (E_y_) electric field on a pyrene-F_4_TCNQ cocrystal. **(B)** Electric-field-dependent ΔM, (ΔM = M(E)-M(E = 0), where M(E) is the value of magnetization under an electric field), inset: perpendicular electric field-dependent magnetization of pyrene-F_4_TCNQ (Reproduced from Yang et al. (2018) with permission from American Chemical Society, Copyright 2018.).

It should be stressed that the materials with anisotropic magnetoelectric coupling properties, which exhibit different energy densities of saturated (or spontaneous) magnetization in different crystal directions (Palneedi et al., 2016), have potential applications in multiferroic memory devices (Spaldin and Ramesh, 2019). Cocrystals have long-range ordered CT networks and largely delocalized π-electrons (Zhu et al., 2021), providing more opportunities for guiding the magnetoelectric coupling of organic materials. Qin et al. have proved the anisotropy of magnetization within C_60_-thiophene between in-plane (easy axis) and out-of-plane (hard axis) directions, which were attributed to the electron-phonon coupling tightly related to the molecular assembly axes and spin cone orientation (Qin et al., 2015b). Latter, Yang et al. obtained pyrene-TCNQ and pyrene-F_x_TCNQ (FxTCNQ, fluorinated derivatives of 7,7,8,8,-tetracyanoquin- odimethane, *X* = 1, 4) cocrystals. They discovered that the higher the CT degree, the better the magnetism. Pyrene-F_4_TCNQ, which had the greatest CT degree, had the best magnetic property and showed the anisotropic magnetoelectric coupling at room temperature. The magnetoelectric coupling coefficient induced by the horizontal electric field was substantially larger than that caused by the perpendicular electric field due to the anisotropic molecular packing and CT interaction in the perpendicular direction (Figures 11A,B) (Yang et al., 2018). This anisotropic magnetoelectric coupling effect of pyrene-F_4_TCNQ met the requirements of perpendicular magnetic recording that could be applied in multiferroic memory devices.

Up to now, extensive studies on magnetic cocrystals have been reported, but some anomalies are still incomprehensible because the internal mechanism is not very clear. Further research into the relationship between the molecular structure and magnetic property is required, which is a challenge for scientists.

## Conclusions and Outlook

This review highlights the advancement in cocrystals with high-performance ambipolar transport, photoelectric conversion, magnetoelectric coupling, and magnetic anisotropy. These materials can not only integrate the properties of the single component but can also exhibit novel characteristics due to the noncovalent intermolecular interaction, such as CT interaction. The inherent advantages of crystals, including lack of defects and no grain boundaries, make it easy to explore the structure-property relationship, facilitating the rational design of cocrystals in OFETs, photoresponse devices, magnetic-field sensors, and magnetic memory devices. However, the development of cocrystals still faces critical challenges: 1) the variety of donors and acceptors for preparing cocrystals is limited, and thus, more suitable materials must be developed; 2) the molecular structure, stoichiometry, and the type of donors and acceptors significantly influence the physical properties of cocrystals, but the specific mechanism is not precise. Selecting D-A molecules to directionally regulate their performance and establishing a complete mechanism are crucial issues in designing organic cocrystals; 3) there still are some problems in large-scale and low-cost preparation methods of organic cocrystals. For practical applications, it is necessary to develop diverse preparation methods for obtaining highly ordered arrays. We believe these difficulties can be overcome with continued research efforts. The cocrystals strategy will play an increasingly critical role in designing organic materials with electronic and magnetic properties.
